# Self-Healing EPDM Rubbers with Highly Stable and Mechanically-Enhanced Urea-Formaldehyde (UF) Microcapsules Prepared by Multi-Step In Situ Polymerization

**DOI:** 10.3390/polym12091918

**Published:** 2020-08-25

**Authors:** Hyeong-Jun Jeoung, Kun Won Kim, Yong Jun Chang, Yong Chae Jung, Hyunchul Ku, Kyung Wha Oh, Hyung-Min Choi, Jae Woo Chung

**Affiliations:** 1 Department of Organic Materials and Fiber Engineering, Soongsil University, 369 Sangdo-ro, Dongjak-gu, Seoul 156-743, Korea; hawllowen1019@gmail.com (H.-J.J.); rjsdnjs7@naver.com (K.W.K.); felnino@naver.com (Y.J.C.); 2 Institute of Advanced Composite Materials, Korea Institute of Science and Technology (KIST), 92 Chudong-ro, Bongdong-eup, Wanju-gun, Jeonbuk 55324, Korea; ycjung@kist.re.kr; 3 Department of Electronic and Communication Engineering, Konkuk University, Seoul 05029, Korea; hcku@konkuk.ac.kr; 4 Department of Fashion, Chung-Ang University, 4726 Seodongdae-ro, Daedeok-myeon, Anseong-si, Gyeonggi-do 17546, Korea; kwhaoh@cau.ac.kr

**Keywords:** dicyclopentadiene (DCPD), ethylene-propylene-diene-monomer (EPDM) rubber, in situ polymerization, microcapsules, self-healing

## Abstract

The mechanically-enhanced urea-formaldehyde (UF) microcapsules are developed through a multi-step in situ polymerization method. Optical microscope (OM) and field emission scanning electron microscope (FE-SEM) prove that the microcapsules, 147.4 μm in diameter with a shell thickness of 600 nm, are well-formed. From ^1^H-nuclear magnetic resonance (^1^H-NMR) analysis, we found that dicyclopentadiene (DCPD), a self-healing agent encapsulated by the microcapsules, occupies ca. 40.3 %(*v*/*v*) of the internal volume of a single capsule. These microcapsules are mixed with EPDM (ethylene-propylene-diene-monomer) and Grubbs’ catalyst via a solution mixing method, and universal testing machine (UTM) tests show that the composites with mechanically-enhanced microcapsules has ca. 47% higher toughness than the composites with conventionally prepared UF microcapsules, which is attributed to the improved mechanical stability of the microcapsule. When the EPDM/microcapsule rubber composites are notched, Fourier-transform infrared (FT-IR) spectroscopy shows that DCPD leaks from the broken microcapsule to the damaged site and flows to fill the notched valley, and self-heals as it is cured by Grubbs’ catalyst. The self-healing efficiency depends on the capsule concentration in the EPDM matrix. However, the self-healed EPDM/microcapsule rubber composite with over 15 wt% microcapsule shows an almost full recovery of the mechanical strength and 100% healing efficiency.

## 1. Introduction

With advances in materials research and technology, efforts to overcome the limitations of existing materials and industries are gradually increasing. There is growing interest in self-healing materials that can enhance the sustainability and utility of polymeric materials which were easily broken by external stimuli [1,2,3]. Self-healing materials are broadly divided into intrinsic and extrinsic systems in terms of the healing mechanism. Among them, the extrinsic self-healing system using microcapsule has many industrial advantages: (1) it can be realized by a simple mixing technique; (2) it possesses excellent self-healing properties [4]; and (3) it can be applied to paints and coating agents in large volumes [5,6,7,8]. Furthermore, it is capable of providing excellent self-healing properties regardless of material’s physical characteristics [9]. In contrast, the intrinsic self-healing system can be realized only when the materials have soft physical properties like rubber [10].

Self-healing microcapsules consist of core healing material capable of a cross-linking reaction and a polymeric shell that can contain and protect the core material [11]. Typically, core self-healing materials are liquid thermosetting substances such as dicyclopentadiene (DCPD) [12,13] and epoxy [14,15]. Thus, when the microcapsules are broken by material’s damage, the healing agents in the microcapsules can easily flow out to the damaged region and then can be polymerized, resulting in the restoration of damaged material’s original morphology and properties [16]. These versatile self-healing microcapsules are generally produced by the in situ polymerization or interfacial polymerization methods. Microcapsules synthesized by the interfacial polymerization method are known to be mechanically stable. However, these microcapsules have the disadvantage of being less efficient to induce self-healing because they include not only self-healing agents but also materials used for shell formation [17]. Despite the relatively lower mechanical stability of the shell, the self-healing microcapsules manufactured by the in situ polymerization method are most commonly used because of the high purity of the contained-core material, which leads to high self-healing efficiency [18].

Among various types of microcapsules prepared by the in situ polymerization method, microcapsules with shells composed of urea-formaldehyde (UF) have mostly been used [19]. White et al. developed a self-healing epoxy by embedding self-healing UF microcapsules and Grubbs’ 1st generation catalyst, and they showed that the fracture load value of the self-healing epoxy recovered to 75% of the original [16]. Wertz et al. manufactured a self-healing poly(lactic acid) with improved crystallinity and heat deflection temperature by introducing UF microcapsules similar in size to the poly(lactic acid) nucleating agent [20]. Furthermore, Song et al. developed the light-induced self-healable coating agent using UF microcapsules for concrete [21]. Nevertheless, as mentioned earlier, these UF microcapsules have exhibited a poor mechanical stability because the shell of the UF microcapsule was very thin compared to the size of the capsule. Due to these limitations, many problems occur, such as easy breakage when introducing a microcapsule into a matrix, or core healing material leaking during storage [22]. To solve these problems, microcapsules having melamine-urea-formaldehyde (MUF) material as a shell component have been proposed; however, these offered limited strengthening of the capsule shell [23]. In addition, thickening the shell to enhance the mechanical stability led to an increase in the size of the capsule, which makes it difficult to adjust the thickness and size of the shell independently [24]. Thus, it is important to develop a method to manufacture the microcapsule with improved mechanical durability and stability for realizing highly efficient self-healing materials, while freely adjusting the size of the microcapsule. Crucially, when introducing self-healing microcapsules into a rubber material which undergoes large deformations such as tension and compression during use, it is necessary to introduce microcapsules with excellent mechanical stability [25].

Considering the impact of rubber materials on the industry, it is anticipated that this study will serve as a cornerstone for the application of self-healing rubber to various industrial fields such as automobile, aviation, space, shipbuilding, et cetera.

## 2. Experimental

### 2.1. Materials

Urea (>98%), ammonium chloride, and *n*-octyl alcohol were all purchased from Daejung Chemicals (Siheung-si, South Korea). Resorcinol was purchased from Junsei Chemical (Tokyo, Japan). Poly(ethylene-alt-maleic anhydride) (EMA), 37 wt% formaldehyde solution, and Grubbs’ 1st generation ruthenium metathesis catalyst were all purchased from Sigma-Aldrich Chemical Co. (Saint Louis, MO, USA). Dicyclopentadiene (95%) was purchased from Acros Organics (Belgium, WI, USA). All reagents were used as received without further purification. Ethylene-propylene-diene-monomer (EPDM) rubber (KeltanÒEco 6950C) was kindly provided by LANXESS (Köln, Germania).

### 2.2. Preparation of Microcapsules

**Figure 1:**
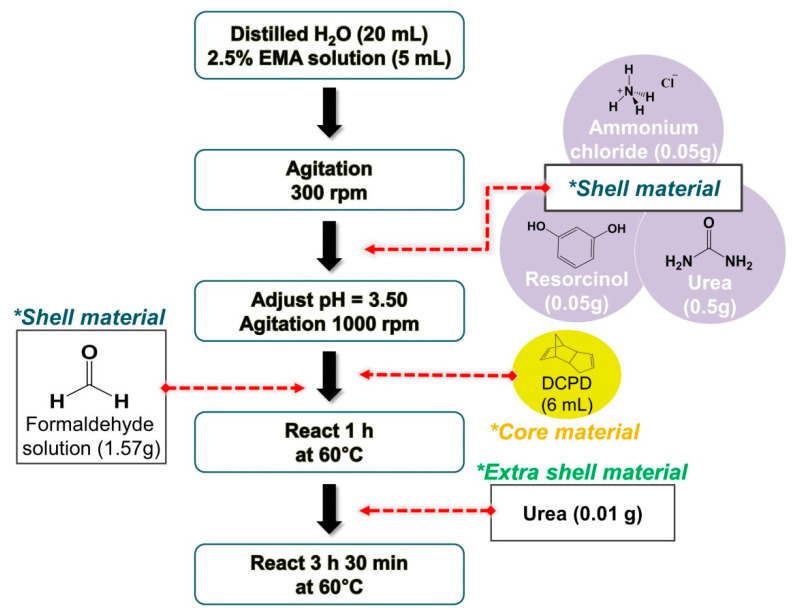
Synthesis flow chart of the mechanically-enhanced urea-formaldehyde (UF) microcapsules using multi-step in situ polymerization.

Mechanically-enhanced microcapsules filled with DCPD were synthesized according to the procedure summarized in Figure 1. First, 20 mL of deionized water and 5 mL of 2.5 wt% aqueous solution of EMA were mixed in a 200 mL beaker. To this aqueous solution, 0.5 g of urea, 0.05 g of ammonium chloride, and 0.05 g of resorcinol were added and dissolved under continuous stirring with a digital mixer (high-speed digital overhead stirrer, HS-100D, DAIHAN Scientific Co., Ltd., Gang-Won-Do, Korea) equipped with a ø 30 mm three-bladed propeller at 300 rpm. After that, the pH of the solution was adjusted to 3.50 by drop-wise addition of sodium hydroxide, and the stirring speed was increased to 1000 rpm. At this stage, one or two drops of 1-octanol were added to eliminate surface bubbles. DCPD was then slowly injected into the solution to form an emulsion and allowed to stabilize for 10 min. The resulting emulsion was heated in a water bath (digital precise water bath, WB-6, DAIHAN Scientific Co., Ltd., Gang-Won-Do, Korea) at a rate of 1 °C/min to the target temperature of 60 °C right after 1.57 g of 37 wt% aqueous solution of formaldehyde was added. After one hour, a further 0.01 g of urea was added to the solution and the reaction proceeded for another 3.5 h under continuous agitation at 1000 rpm. Then, the mixer and hot plate were switched off, and the suspension of microcapsules was cooled to ambient temperature. After cooling, the microcapsules were isolated using a separation funnel and washed with deionized water and ethanol under vacuum with a coarse-fritted filter and allowed to air dry over 24 h. The preparation procedure is presented in Figure 1.

### 2.3. Preparation of Self-Healing EPDM/Microcapsule Rubber Composite

The EPDM matrix (7 g) was dissolved in chloroform (80 mL) and degassed by ultrasonication. Grubbs’ 1st generation ruthenium metathesis catalyst (2.5 wt%) and the desired amount of DCPD-filled microcapsules (0, 1, 5, 10, 15, 20 wt%) were added to the EPDM solution. The mixture was thoroughly mixed and poured into a Teflon-coated glass. After 36 h, the cast films were removed and dried at room temperature for 48 h. Manufactured EPDM/microcapsule rubber composite’s compositions and sample names are outlined in Table 1.

### 2.4. Characterization

The formation of the prepared microcapsules was confirmed with an optical microscope (OM, BX53M, Olympus, Tokyo, Japan). Obtained optical images were then processed with ImageJ software to determine the average size and size distribution of the microcapsules. The surface morphology and the thickness of the shell of the microcapsules were analyzed using a field emission scanning electron microscope (FE-SEM, GeminiSEM 300, Carl Zeiss, Oberkochen, Germania). The presence of self-healing substances in the microcapsules was confirmed by ^1^H-nuclear magnetic resonance (^1^H-NMR, FT-NMR 400MHz, Bruker Advance, Billerica, MA, USA) analysis. For this, a few microcapsules were placed in the CDCl_3_ NMR solvent (5 mL) and ruptured. The ruptured microcapsules were filtered using a PTFE syringe filter with a pore size of 0.45 µm, and ^1^H-NMR measurement was performed on the filtrate solution. To calculate the amount of the self-healing agents inside the microcapsules, the weight of the microcapsules containing DCPD and microcapsules without DCPD was compared as follows (Equation (1)).

(1) $$\mathrm{Core}\;\mathrm{content}\;\left(\%\right)=\;\frac{Wv-Wd}{Wv}\times 100$$

where *W_v_* is the initial weight of the capsules before self-healing agent removal and *W_d_* is the weight of the capsules following self-healing agent removal. To remove the self-healing agents from the microcapsules, the microcapsules containing the self-healing agents were thoroughly crushed with a mortar and pestle and washed with acetone. After that, the filter funnel was used to collect the broken microcapsules, and then dried in a vacuum oven at 60 °C for 24 h.

The extent to which the encapsulated core material occupies the cavity of the microcapsule, namely encapsulation capability (%(*v*/*v*)), was also evaluated using the molar ratio obtained from the ^1^H-NMR analysis. The whole process proceeded in three stages. First, the amount of self-healing agent and the resulting molar ratio (self-healing agent/reference compound) were plotted to create a 5-point calibration curve. For this, DCPD (10, 30, 50, 70, 90 µL) was dissolved in a fixed amount of CDCl_3_ NMR solvent (2 mL) and benzene (10 µL), which was used as a reference substance. Then, by using the integrated peak area obtained by NMR software, the molar ratios of added DCPD and benzene were calculated as follows (Equation (2)).

(2) $$M_{DCPD/benzene}=\frac{I_{DCPD}}{I_{benzene}}\times \frac{H_{benzene}}{H_{DCPD}}$$

where $M_{DCPD/benzene}$ is the molar ratio of added DCPD and benzene, $I_{DCPD}$ is the integrated peak area corresponding to DCPD, $I_{benzene}$ is the integrated peak area corresponding to benzene, and $H_{benzene}$ and $H_{DCPD}$ are the number of contributing protons of observing NMR peaks for benzene and DCPD, respectively. Second, the amount of core material contained in a single microcapsule was calculated, assuming that the core material was evenly distributed with the same amount in each capsule. Then, 0.05 g of microcapsules were taken and put into a solution consisting of the same amount of CDCl_3_ NMR solvent and benzene as in the first step, and thoroughly ground. The broken microcapsules were removed using a PTFE syringe filter with a pore size of 0.45 µm, and ^1^H-NMR analysis was performed on the obtained filtrate solution. The molar ratio (DCPD/benzene), which was calculated from Equation (2), was introduced into the calibration curve so that the amount of DCPD in 0.05 g of microcapsules could be determined. Furthermore, the amount of DCPD encapsulated in a single microcapsule was determined by utilizing the weight of one microcapsule measured with a microbalance (Microbalance, M3P, Sartorius, Göttingen, Germania) was 8.33 ± 1.53 × 10^−3^ mg. For reliable data, all measurements were performed five times, and the average value of the values excluding the maximum and minimum values was taken. Finally, the encapsulation capability was calculated from the ratio of the amount of self-healing agent, which was calculated in the second stage, to the internal volume of the microcapsule, in other words, the amount of self-healing agent that can be encapsulated in a single capsule at the maximum. In this case, the capsules were assumed to have a perfectly spherical shape.

The self-healing behavior of EPDM/microcapsule rubber composite was verified using OM and Fourier-transform infrared spectroscopy (FT-IR, vertex 70, Bruker, Billerica, MA, USA). For this, the composite film having a thickness of 8.5 ± 1.2 mm was prepared, and a razor blade was used to scratch the surface of the rubber. After five hours following the surface damage, the change in the damaged morphology was observed through an OM. The FT-IR analysis of the DCPD monomer using the KBr pellet technique and the self-healed composite using the attenuated total reflectance (ATR) method was employed to confirm the ring-opening metathesis polymerization reaction at damaged site.

Mechanical properties of the self-healing EPDM/microcapsule rubber composites were measured using a universal testing machine (UTM, DR-100, Dr-tech, Gyeonggi-do, Korea), and tensile and tear tests were carried out based on ASTM D 638-V and ASTM D 624 standard test method, respectively. The thickness of the test specimens was about 0.85 ± 0.12 cm, and the measurements were conducted five times at room temperature using a 10 kgf load cell with a cross-head speed of 500 mm/min. The data closest to the average of the values excluding the maximum and minimum value was taken. In addition, by comparing the value of the mechanical properties of the manufactured EPDM composite (virgin) to the healed EPDM composite, which was completely cut in half and the cut surface was immediately contacted for 5 h, self-healing efficiency was calculated with the following equation (Equation (3)).

(3) $$\eta \;\left(\%\right)=\frac{Healed\;comopsite’s\;mechanical\;property}{Virigin\;composite’s\;mechanical\;property}\times 100$$

where η (%) represents the self-healing efficiency. The mechanical properties of the *Healed* and *Virgin* composites are the value of the stress in the tensile test and the tear strength in the tear test, respectively.

## 3. Results and Discussion

**Figure 2:**
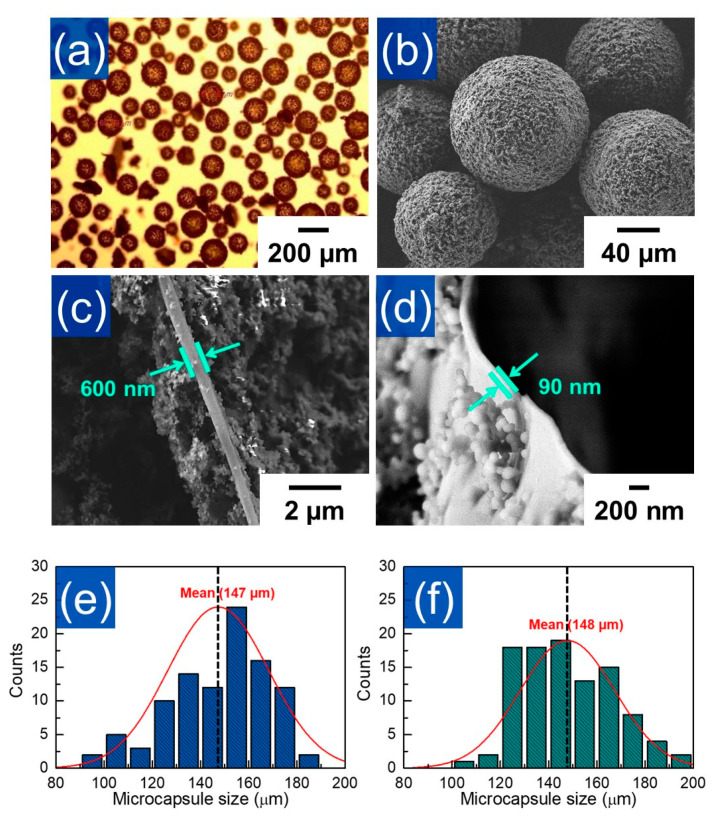
(**a**) Optical microscopy and (**b**) FE-SEM images of the mechanically-enhanced UF microcapsules. The shell thickness FE-SEM images of (**c**) mechanically-enhanced UF microcapsules and (**d**) conventional UF microcapsules. The size distributions of (**e**) mechanically-enhanced and (**f**) conventionally prepared UF microcapsules. Standard normal distribution curves are superimposed on the histogram of the samples.

As shown in Figure 2a,e, it was observed that microcapsules with an average size of about 147 µm were well-formed in a spherical shape. The microcapsules had a rough and uneven surface (Figure 2b), which is a common feature found in UF microcapsules [12].

Especially, the microcapsules prepared from the multi-step in situ polymerization method had a rougher shell surface than the conventional UF microcapsules. In addition, it was found that the shell thickness of microcapsules synthesized by the multi-step in situ polymerization method was about 600 nm (Figure 2c), while the thickness of the same size UF microcapsule which was prepared by the conventional method was about 90 nm (Figure 2d). These results indicated that the shells were secondarily re-grown on the surface of the microcapsules by the subsequent addition of extra urea after preferentially formed microcapsules into the target size. Thus, it was thought that UF microcapsules with enhanced shell thickness can be successfully produced through a multi-step in situ polymerization method without affecting the capsule size. (Figure 2e,f)

**Figure 3:**
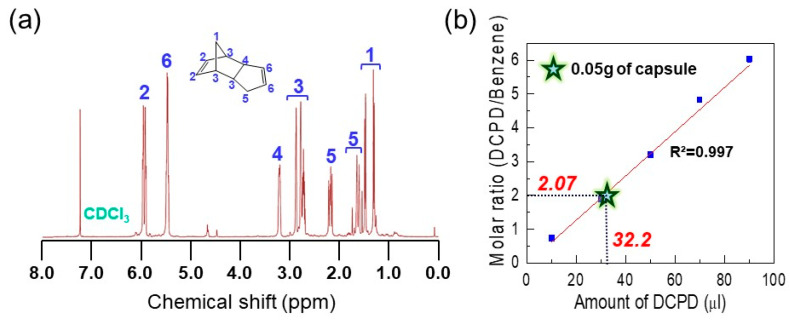
(**a**) ^1^H-NMR spectrum of the extracted dicyclopentadiene (DCPD). (**b**) The calibration line of the DCPD/benzene molar ratio with function of the known volume of DCPD (10, 30, 50, 70, 90 µL). The solid line represents the result of a least squared linear fit with R^2^ = 0.997. Star-shaped mark represents the DCPD/benzene molar ratio value measured from 0.05 g of mechanically-enhanced microcapsules and corresponding amount of DCPD.

^1^H-NMR analysis was carried out to verify the successful encapsulation of the core material. As shown in Figure 3a, the NMR spectrum of material extracted from the microcapsules was the same as that of neat DCPD, suggesting that the healing agent was stably preserved in the microcapsules without any chemical deformation during the encapsulation process. From weight change of the microcapsule before and after self-healing agent removal (Equation (1)), it was calculated that the core content of the microcapsules was ca. 70% (*w*/*w*). In addition, we made the DCPD calibration curve using benzene as a reference substance (Equation (2)) and calculated the encapsulation capability of a single microcapsule. As shown in Figure 3b, it was observed that 0.05 g of microcapsules included 32.2 mL of DCPD. When considering the mean diameter (ca. 147 mm) of microcapsule and thickness (600 nm) of microcapsule shell (Figure 2), we found that ca. 40.3% (*v*/*v*) of DCPD was charged in the single microcapsule.

**Figure 4:**
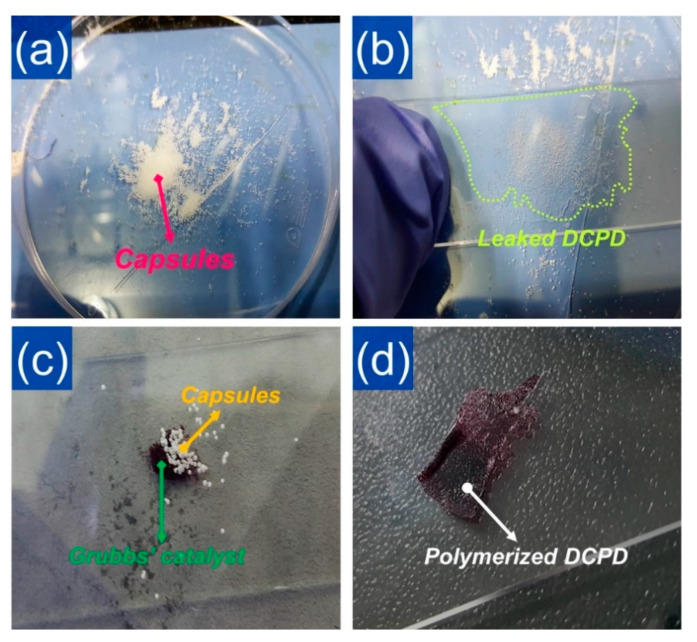
Photo images of (**a**) the prepared powder-like mechanically-enhanced microcapsules, (**b**) the liquid DCPD monomer leaked from the ruptured microcapsules, (**c**) the mixture of the microcapsules and the Grubbs’ catalyst, and (**d**) the solid DCPD formed after 5 h of the reaction between the leaked DCPD and the catalyst.

The self-healing phenomenon was preliminarily tested before manufacturing the self-healing composite. First, microcapsules were placed between two glass covers and the glass covers were pressed to break the capsules. As shown in Figure 4a,b, it was observed that the liquid DCPD stored inside the capsules was released after breaking the microcapsules. The same procedure was also employed in the mixture of the capsules and Grubbs’ catalyst (Figure 4c). As shown in Figure 4d, a hard substance was formed on the cover glass after 5 h following the capsule breaking. It is well known that the cross-linking reaction of the DCPD, namely, ring-opening metathesis polymerization (ROMP) reaction, occurs when DCPD meets the Grubb’s catalyst. Thus, the formation of a solid substance observed in Figure 4d clearly indicated that the DCPD that came out of the broken microcapsules underwent a ROMP reaction with the catalyst, resulting in the polymerized DCPD with a thermosetting cross-linking structure [26,27,28,29,30,31]. From these results, it is expected that when the EPDM/microcapsule rubber composite is damaged, DCPD will flow out to the damaged site from the ruptured microcapsules and form the crosslinked DCPD at the damaged site by the ROMP reaction with the catalyst, implying that EPDM/microcapsule rubber composites can act as a smart self-heal rubber system.

**Figure 5:**
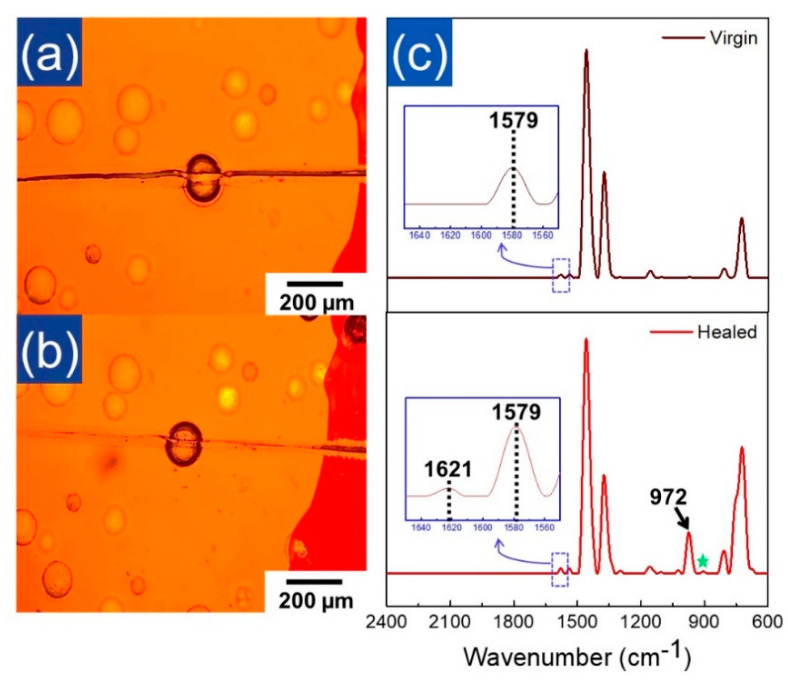
Optical microscopy images of the EPDM/microcapsule rubber composites with 10 wt% of the mechanically-enhanced microcapsules (**a**) right after being scratched with a razor blade and (**b**) after 5 h following scratching. (**c**) Normalized ATR FT-IR spectra of the neat EPDM rubber (top) and the healed EPDM/microcapsule rubber composite (bottom). ATR FT-IR spectrum of the healed EPDM/microcapsule rubber composite measured at the damaged crack region after 5 h following scratching with razor blade.

To investigate the self-healing behavior of the EPDM/microcapsule rubber composite, we notched the surface of the EPDM/microcapsule rubber composite with 10 wt% of microcapsules using a razor blade and observed the notched region by OM. Figure 5a showed that a notched crack was clearly formed at a certain depth. After 5 h, however, the notch was faded and overall filled with a new substance (Figure 5b). This suggested that the DCPD flowed out to the damaged site of the composites from the broken microcapsules and the DCPD that had filled the notched crack was cross-linked by catalyst, as mentioned above. To verify whether the filling of the notched crack was induced by the cross-linking reaction of DCPD, we observed the IR spectrum of the damaged notch region of EPDM/microcapsule rubber composite after 5 h following the damage using ATR-FT IR.

As shown in Figure 5c (top), the virgin EPDM showed IR bands at 1155 cm^−1^, 1375 cm^−1^, and 1458 cm^−1^ corresponding to the –CH_2_ scissoring and –CH_3_ stretching vibration of the ethylene-propylene unit of EPDM, respectively. The peaks corresponding to the C–H bending vibration of the double bond of the ethylidene norbornene (ENB) unit of virgin EPDM were also observed at 725 cm^−1^ and 808 cm^−1^ [32]. On the other hand, as shown in Figure 5c (bottom), the IR spectrum observed at the crack region showed the newly developed strong band at 972 cm^−1^ corresponding to the characteristic peak of the crosslinked DCPD, while the unreacted DCPD were little observed (star mark at 906 cm^−1^ in the Figure 5c (bottom)) (Figure S1 in supporting information) [16]. Furthermore, the small peak corresponding to the cross-linked DCPD was also observed at 1621 cm^−1^ (Figure S1 in supporting information) [33]. These clearly indicated that the liquid DCPD that had been flowed toward the notched crack changed into the cross-linked DCPD (PDCPD) at the damaged crack part. Thus, it was thought that the mechanically-enhanced UF microcapsules produced by the multi-step in situ polymerization successfully provided the EPDM/microcapsule rubber composites with the self-healing property.

**Figure 6:**
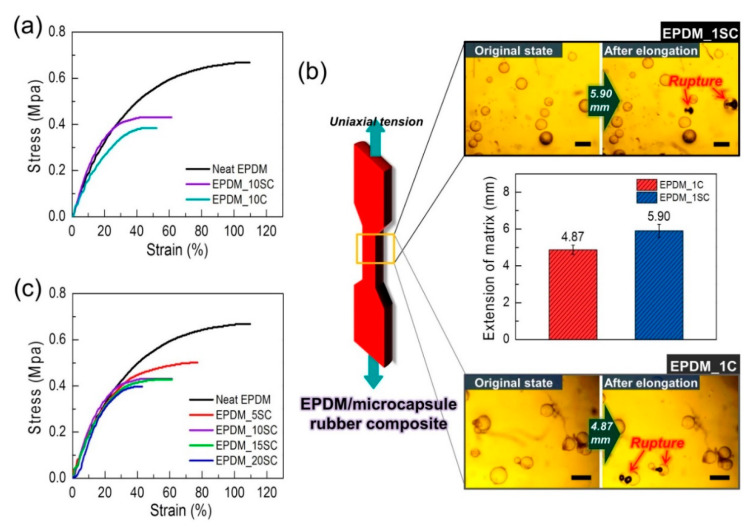
(**a**) The stress–strain curves of neat EPDM rubber, the EPDM/microcapsule rubber composites with 10 wt% of mechanically-enhanced UF microcapsules (EPDM_10SC), and the EPDM/microcapsule rubber composites with 10 wt% of conventionally prepared UF microcapsules (EPDM_10C). (**b**) The required extension value of the EPDM/microcapsule rubber composites to break two capsules in the composite (scale bars: 200 µm). (**c**) The stress-strain curves of the EPDM/microcapsule rubber composites with varied mechanically-enhanced UF microcapsule contents.

Two types of EPDM rubber composites with the mechanically-enhanced UF microcapsules produced by multi-step in situ polymerization and the typical UF microcapsules produced by conventional in situ polymerization were individually manufactured, and their mechanical properties were analyzed using UTM. Figure 6a is the stress-strain curves of neat EPDM, EPDM rubber composites with 10 wt% of the mechanically-enhanced UF microcapsules (EPDM_10SC), and EPDM rubber composites with 10 wt% of the conventional UF microcapsules (EPDM_10C). Figure 6a depicts that EPDM rubber composite incorporated with mechanically-enhanced microcapsule had a toughness value of about 47% higher than that with the conventional one. This was attributed to the improvement of the mechanical stability of UF microcapsules with thicker shells relative to the conventional UF microcapsules. In addition, the rough surface of the UF microcapsules formed by the multi-step in situ polymerization might provide the mechanical locking effect between the microcapsule and EPDM rubber [15]. For a more detailed analysis of the stability of the capsules, we compared the rupture behavior of microcapsules in the EPDM/microcapsule rubber composites with 1 wt% of the mechanically-enhanced UF microcapsules (EPDM_1SC) and 1 wt% of the conventional UF microcapsules (EPDM_1C) during the tensile elongation. As shown in Figure 6b, the mechanically-enhanced UF microcapsules were stable until about 5.90 mm elongation, whereas the conventional UF microcapsules began to break when the extension of the composite was about 4.87 mm. This suggested that the mechanical stability of the microcapsules affected the mechanical properties of the EPDM/microcapsule rubber composites. Thus, it was thought that the multi-step in situ polymerization was effective in strengthening the mechanical stability of the microcapsule and further improving of the mechanical properties of EPDM/microcapsule rubber composites. Unfortunately, EPDM/microcapsule rubber composites showed lower mechanical properties than the neat EPDM rubber, and the tensile strength of the composite decreased from ca. 0.50 MPa to ca. 0.40 MPa as the content of the capsules in the rubber composites increased. (Figure 6c) This may be associated with the fact that micro-sized capsules that contained a liquid core agent acted as a mechanical defect of the rubber composites. Moreover, interfacial heterogeneity between EPDM rubber and microcapsules may cause a decrease in the mechanical properties of the EPDM/microcapsule rubber composites [4,20,34,35]. Indeed, we confirmed that such limitation of microcapsule can be sufficiently compensated through the reduction of the capsule size and surface modification (Figure S2 in supporting information). The effect of capsule size and surface modification on the mechanical properties of rubber/microcapsule composites will be discussed in a subsequent paper since the scope of the current study deals with how microcapsules with mechanically-enhanced shells can have positive effects on the EPDM rubber composites compared to conventional UF microcapsules.

**Figure 7:**
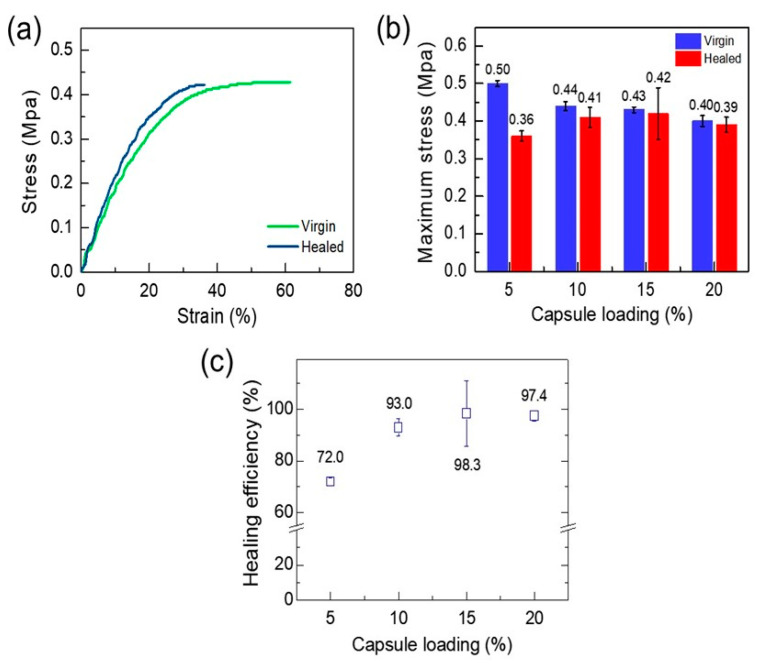
(**a**) Stress–strain curves of the virgin and the healed EPDM/microcapsule rubber composites with 15 wt% of the mechanically-enhanced UF microcapsules. (**b**) The maximum stress values of the virgin and the healed EPDM/microcapsule rubber composites. (**c**) The self-healing efficiencies of the EPDM/microcapsule rubber composites with different amounts of the mechanically-enhanced UF microcapsules.

Figure 7a displays the stress–strain curves of the virgin and self-healed EPDM/microcapsule rubber composites with 15 wt% of microcapsules. Although the strain value of the healed EPDM/microcapsule rubber composite has fallen compared to that of the virgin EPDM/microcapsule rubber composite, the value of the maximum stress was almost the same, indicating that complete self-healing had occurred. In addition, the healed EPDM/microcapsule rubber composites with 10 and 20 wt% microcapsules also exhibited stress values not differing greatly with those of their virgin samples, excepting for 5 wt% microcapsule loading (Figure 7b and Figure S3 in supporting information).

The calculated self-healing efficiency of the EPDM/microcapsule rubber composites with 5, 10, 15, and 20 wt% microcapsule loading were 72.0, 93.0, 98.3, and 97.4%, respectively (Figure 7c). These results revealed that the wounded EPDM/microcapsule rubber composites fully restored their intrinsic mechanical strength by the self-healing reaction when the composites included the microcapsule over 10 wt%.

**Figure 8:**
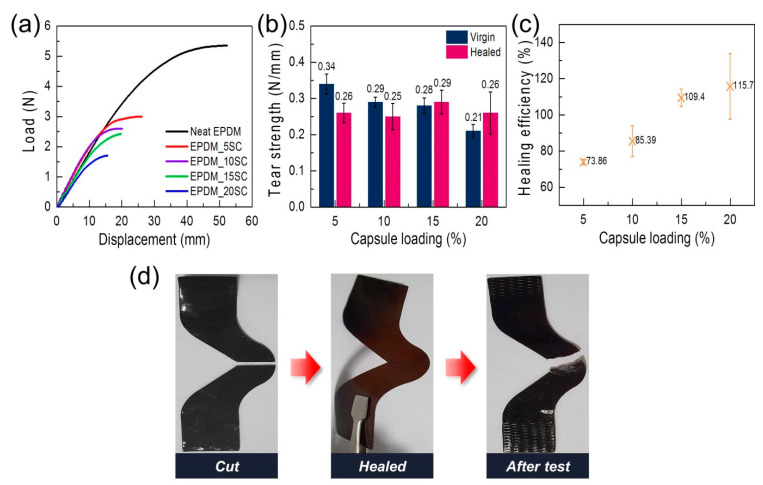
(**a**) Load-displacement curves of the EPDM/microcapsule rubber composites with varied amounts of the mechanically-enhanced UF microcapsules. (**b**) The tear strength values of the virgin and the healed EPDM/microcapsule rubber composites with varied amounts of mechanically-enhanced UF microcapsules. (**c**) The self-healing efficiencies of the EPDM/microcapsule rubber composites with varied amounts of mechanically-enhanced UF microcapsules based on the recovery of the tear strength. (**d**) The photo images of the cut and the healed EPDM_15SC, and the photo image of the EPDM_15SC torn by the tear test after the healing.

In addition to the tensile strength, we measured the tear strength of the EPDM/microcapsule rubber composites with a varied amount of microcapsules. As shown in Figure 8a, the incorporation of capsules reduced the original tear properties of the EPDM rubber, and the maximum load and maximum displacement decreased as the microcapsules content increased. This could be attributable to the structural limitation that the mechanical properties of the microcapsules are entirely dependent on the severely thin shell relative to its size, and the heterogeneity of the interface with the matrix rubber. Figure 8b,c present the tear strength values and tear strength-based healing efficiencies of virgin and healed EPDM/microcapsule rubber composites. Their healing efficiencies were increased by increasing the microcapsule loading, and even the healing efficiencies of the composites were greater than 100% over 15 wt% microcapsule content. It has been reported that if self-healing occurs perfectly, the crack generated after self-healing can initiate not only at the original crack position but also at the new location [36]. Indeed, we observed that the tearing of the healed EPDM/microcapsule rubber composite with 15 wt% microcapsule contents occurred along a different path with the original crack. (Figure 8d), resulting in longer tear path and higher healing efficiency. (Figure S4 and movie S1 in supporting information). This result clearly showed that the damage of the EPDM/microcapsule rubber composite was perfectly healed.

## 4. Conclusions

In this study, the mechanically-enhanced UF microcapsules with liquid DCPD self-healing core agent were prepared through the multi-step in situ polymerization and possessed independently controlled size and shell thickness. Although the EPDM/microcapsule rubber composites possessed lower mechanical properties than the neat EPDM rubber, the EPDM/microcapsule rubber composites with the mechanically-enhanced UF microcapsules showed more stable mechanical properties than the EPDM/microcapsule rubber composites with similar-sized UF microcapsules prepared by conventional method. This may be attributed to the thicker shell structure of the mechanically-enhanced UF microcapsules. When the EPDM/microcapsule rubber composites were damaged, DCPD flowed out to the damaged site of the composites from the broken microcapsules, filled the notched crack, and was cross-linked by catalyst. As a result, the EPDM/microcapsule rubber composites with the mechanically-enhanced UF microcapsules showed highly efficient self-healing properties against the tensile and tear deformations of the composites. Therefore, rubber composites with the mechanically stable microcapsules can give rise to sustainable rubber material that can endure numerous stimuli and deformations.

## Figures and Tables

**Table 1:** Compositions and sample names of the ethylene-propylene-diene-monomer (EPDM)/microcapsule rubber composites.

Sample	Conventional Capsule	Mechanically- Enhanced Capsule	Microcapsule Loading (wt%)	Grubbs’ Catalyst (wt%)
Neat EPDM	-	-	-	-
EPDM_1SC	-	O	1	2.5
EPDM_1C	O	-	1	2.5
EPDM_5SC	-	O	5	2.5
EPDM_10SC	-	O	10	2.5
EPDM_10C	O	-	10	2.5
EPDM_15SC	-	O	15	2.5
EPDM_20SC	-	O	20	2.5

## Supplementary Materials

The following are available online at https://www.mdpi.com/2073-4360/12/9/1918/s1. Figure S1: Normalized absorption spectra of the neat DCPD, the polymerized DCPD (PDCPD), and the catalyst; Figure S2: Tensile stress-strain curves of rubber composites embedded with 80 μm size microcapsules: (a) the neat rubber, (b) the rubber composite with 5wt% capsules, (c) rubber composite with 1wt% capsules, (d) rubber composite with 1wt% surface-modified capsules; Figure S3: Tensile stress-strain curves of the virgin and the healed EPDM/microcapsule rubber composites with the varied amount of the mechanically-enhanced UF microcapsules: (a) EPDM_5SC, (b) EPDM_10SC, and (c) EPDM_20SC; Figure S4: Load-displacement curves of the virgin and the healed EPDM/microcapsule rubber composites with the varied amount of the mechanically-enhanced UF microcapsules: (a) EPDM_5SC, (b) EPDM_10SC, (c) EPDM_15SC, and (d) EPDM_20SC; Movie 1: Tensile tear test of the healed EPDM/microcapsule rubber composite.
