# Supplementary material for: Self-Healing EPDM Rubbers with Highly Stable and Mechanically-Enhanced Urea-Formaldehyde (UF) Microcapsules Prepared by Multi-Step In Situ Polymerization
Source: Polymers (Basel). 2020 Aug 25;12(9):1918. doi: 10.3390/polym12091918 (PMC7565145; doi:10.3390/polym12091918)
Supplement: Supplementary file 1 [file polymers-12-01918-s001.pdf]

Supplementary Materials:

Self-Healing EPDM Rubbers with Highly Stable and Mechanically-Enhanced Urea-Formaldehyde (UF) Microcapsules Prepared by Multi-Step In-Situ Polymerization

Hyeong-Jun Jeoung[1], Kun Won Kim[1], Yong Jun Chang[1], Yong Chae Jung[2], Hyunchul Ku[3], Kyung Wha Oh[4], Hyung-Min Choi[1,] and Jae Woo Chung[1,*]*

[1]Department of Organic Materials and Fiber Engineering, Soongsil University, 369 Sangdo-ro, Dongjak-gu, Seoul 156-743, South Korea

[2] Institute of Advanced Composite Materials, Korea Institute of Science and Technology (KIST), 92 Chudong-ro, Bongdong-eup, Wanju-gun, Jeonbuk, 55324, South Korea

[3]Department of Electronic and Communication Engineering, Konkuk University, Seoul 05029, South Korea

[4]Department of Fashion Design, Chung-Ang University, 4726 Seodongdae-ro, Daedeok-myeon, Anseong-si, Gyeonggi-do 17546, South Korea

[*]Corresponding Authors:

J. W. Chung; Tel: +82-2-828-7047; Fax: +82-2-817-8346; e-mail: jwchung@ssu.ac.kr

H.-M. Choi; Tel.: +82-2-820-0626; Fax: +82-2-817-8346; e-mail: hchoi@ssu.ac.kr

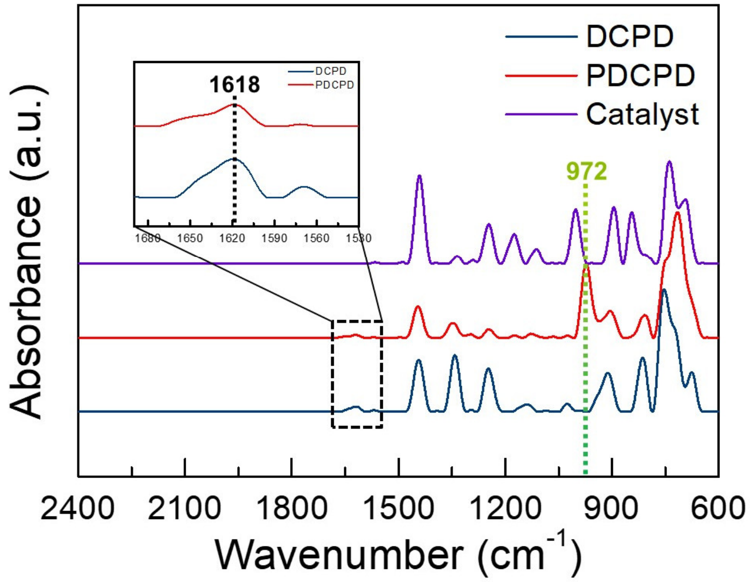

Figure S1. Normalized absorption spectra of the neat DCPD, the polymerized DCPD (PDCPD), and the catalyst.

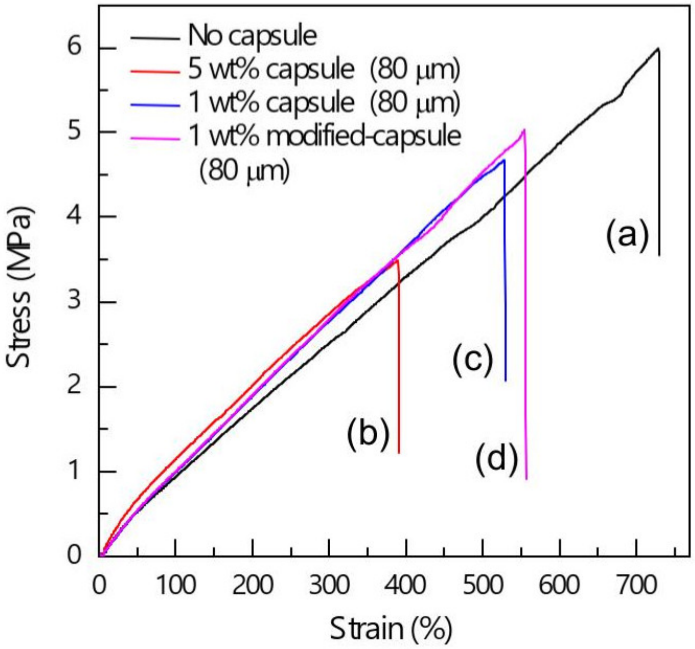

Figure S2. Tensile stress-strain curves of rubber composites embedded with 80 μm size microcapsules: (a) the neat rubber, (b) the rubber composite with 5wt% capsules, (c) rubber composite with 1wt% capsules, (d) rubber composite with 1wt% surface-modified capsules.

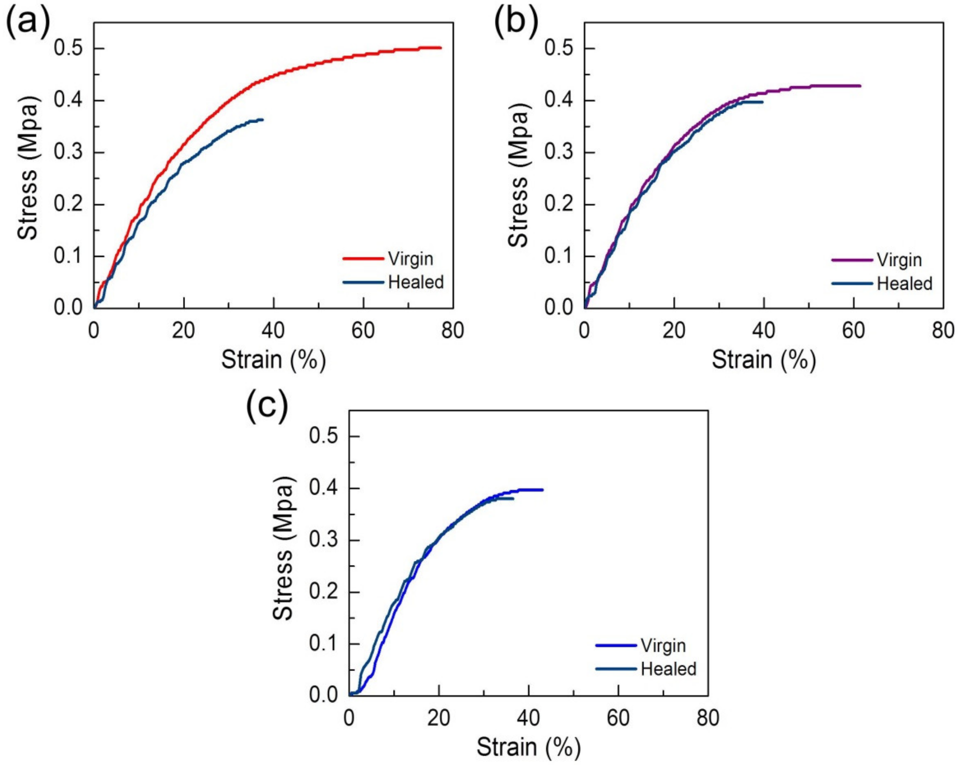

Figure S3. Tensile stress-strain curves of the virgin and the healed EPDM/microcapsule rubber composites with the varied amount of the mechanically-enhanced UF microcapsules: (a) EPDM_5SC, (b) EPDM_10SC, and (c) EPDM_20SC.

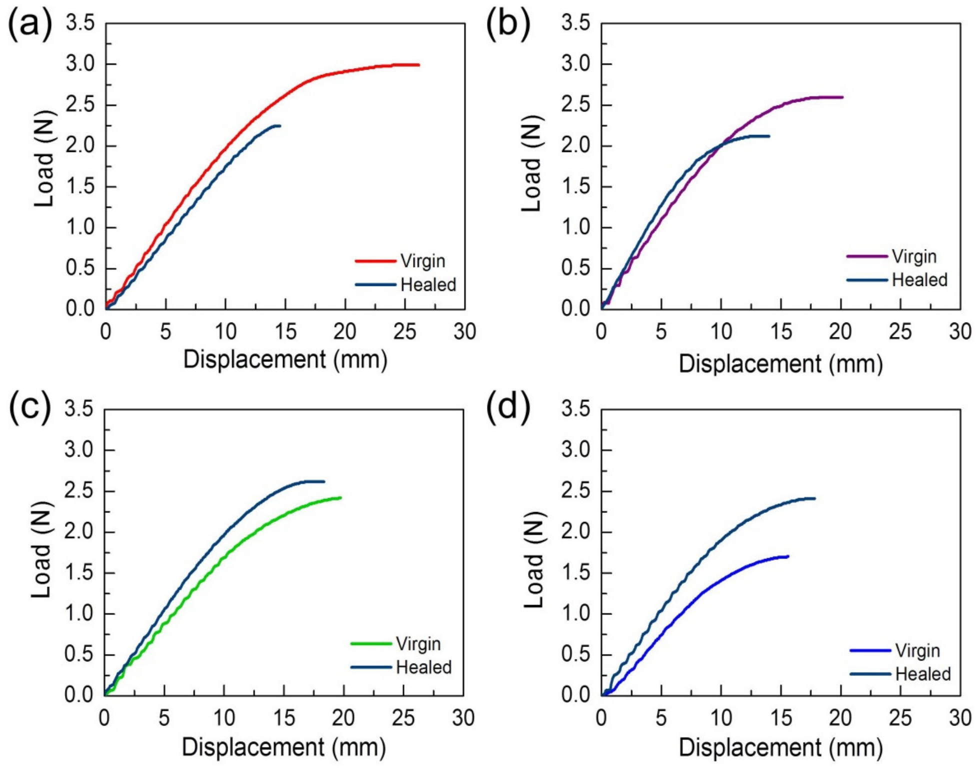

Figure S4. Load-displacement curves of the virgin and the healed EPDM/microcapsule rubber composites with the varied amount of the mechanically-enhanced UF microcapsules: (a) EPDM_5SC, (b) EPDM_10SC, (c) EPDM_15SC, and (d) EPDM_20SC.
